# Epidemiology of uveal melanoma in Brazil

**DOI:** 10.1186/s40942-020-00261-w

**Published:** 2020-11-11

**Authors:** Evandro Lucena, Daniel Cohen Goldemberg, Luiz Claudio Santos Thuler, Andreia Cristina de Melo

**Affiliations:** Clinical Research Division, Brazilian National Cancer Institute–COPQ/INCA, Rua André Cavalcanti, 37-50 Andar – Anexo, Centro, Rio de Janeiro, RJ 20231-050 Brazil

## Abstract

**Purpose:**

To report the prevalence of uveal melanoma in a Hospital database in Brazil over the period of 16 years (2000 to 2016).

**Design:**

Descriptive epidemiological study evaluating the Brazilian Hospital Based Cancer Registries.

**Participants/methods:**

Uveal melanomas were identified based on ICD-O-3 codes C69.3 [choroid], C69.4 [ciliary body and iris], and C69.2 [retina]) derived from the Integrator Registry database. Kolmogorov–Smirnov Test was used for evaluation of normality of data, t-test and Chi square were used for categorical and continuous variables respectively using SPSS Software.

**Main outcome measures:**

Age, sex, education, regional distribution, clinical staging at the diagnosis, time from diagnosis to treatment (≤ 60 days versus > 60 days) and first-course therapy (surgery, chemotherapy, radiotherapy or a combination of such).

**Results:**

There were 2166 cases of uveal melanoma representing 5.4% of all cases of melanoma. Histological confirmation of uveal melanoma was available in all cases. Higher prevalence of 1139 cases (52.6%) in women than 1027 cases (47.4%) in men was observed. Age distribution revealed 1411 cases (65.1%) in the group between 41 and 69 years old. A total of 429 (19.8%) patients were classified as initial disease and 334 (15.4%) as advanced (regional or distant metastases). Staging as initial disease was more frequent (113–24.8%) in patients with > 8 school years than in patients with < 8 school years (179–17.6%) reflecting disparities in healthcare access between those two populations. No difference was noticed in terms of diagnosis, staging and treatment after the Brazilian “60 days law” (Federal Law 12.732/12) came into effect in 2013 regulating the maximum period that a patient with cancer has to wait until start the treatment.

**Conclusion:**

Epidemiological data is critical for planning early treatment strategies and allocating medical resources. This study intended to understand the characteristics of uveal melanoma in Brazil.

## Introduction

Uveal melanoma is the most common primary intraocular malignancy and represents approximately 5% of all melanoma cases [1]. The uvea is composed by the middle vascular pigmented layer of the ocular globe, which encompasses iris, ciliary body and choroid [2–4]. A heterogeneous incidence of 4.3 to 10.6 cases of uveal melanoma per million of people is verified each year globally depending on the studied population, inclusion criteria and methodology used for the study [4]. Although the disease has no sex preference, it is more common in the middle aged Caucasian population. Presence of choroidal nevus, certain skin conditions such as dysplastic nevus syndrome and nevus of Ota are the most recognized risk factors [1]. Even though UV exposure has been theorized to increase the risk for developing uveal melanoma, it has not been effectively proven [3]. A global trend to increased incidence in this particular type of cancer has been observed by some investigators, though this is a matter of discussion [3–5].

Epidemiological studies in the Brazilian population are mostly related with skin melanoma and mucosal melanoma [6, 7] and data for uveal melanomas are scarce and inconsistent. The adjusted cutaneous melanoma estimated incidence of 4200 cases/year for males and 4250 cases/year for females was recently published by the Brazilian National Cancer Institute for the year of 2020 [8]. Higher incidence is observed in the southern states of Brazil and a trend for increased incidence is observed but studies suggest more data is necessary for tracing strategies for better public health management and understanding the epidemiology of the disease [6, 9, 10].

Uveal melanoma is associated with abnormal proliferation of uveal melanocytes. Different histological subgroups are observed with more aggressive behavior and systemic metastases. Cytogenetic aspects such as chromosome 3 monosomy and the presence of mutation in the *GNAQ* and *BAP1* genes are also related with higher aggressiveness and systemic spread [11] and are object of research worldwide. Recently, *PALB2* and *MLH1* germline mutations were described to present moderate evidence of hereditary predisposition and several other candidate susceptibility genes also suggested association with uveal melanomas [12].

Clinical presentation of uveal melanoma depends on the tumor location within the uveal tissue, size, pigmentation, associated bleeding, serous retinal detachment, inflammation and extra scleral extension. Most patients complain of decreased visual acuity and blurred vision symptoms [4, 5, 13].

Even with the most modern treatment techniques the visual prognosis is usually poor and many patients develop functional blindness in the affected eye. Metastization to liver and other organs is a common cause of death despite recent advances in medical therapy and the overall survival is unchanged over the last 70 years [5, 14, 15].

The aim of this study was to analyze epidemiological data in patients with diagnosed of uveal melanoma with an emphasis on prevalence data in Brazil.

## Methods

### Population study

A descriptive epidemiological study was carried out evaluating data from the Brazilian hospital-based cancer registries system obtained through the Integrator System (Brazilian National Cancer Institute—INCA, available at https://irhc.inca.gov.br/RHCNet/ visualizaTabNetExterno.action) and the Oncocenter Foundation of São Paulo, also available on the internet (Oncocentro Foundation, https://200.144.1.68/cgi-bin/dh?rhc/rhc-geral.def) between 2000 and 2016 which included available data from 25 states and the Federal District of Brazil. The database included information from 321 hospitals countrywide.

### Data analysis

The following variables were analyzed in the study: sex, age, school education (years of formal education < 8 years = less than elementary and ≥ 8 years = more than elementary formal education), geographic region of the registry hospital, year of the diagnosis, clinical staging at the diagnosis (initial: stages I and II – Melanoma restricted to the eye, without extraocular extension and measuring up to 15.0 mm thickness and up to 18.0 mm in the largest basal diameter versus Advanced: stages III – Melanoma of any size with extraocular involvement and IV—lymph node metastasis and/or distant metastasis, based on TNM classification of malignant tumors and AJCC Stage groups) [16], time from diagnosis to treatment (≤ 60 days versus > 60 days), first-course therapy (surgery, chemotherapy, radiotherapy or a combination of such).

**Fig. 1:**
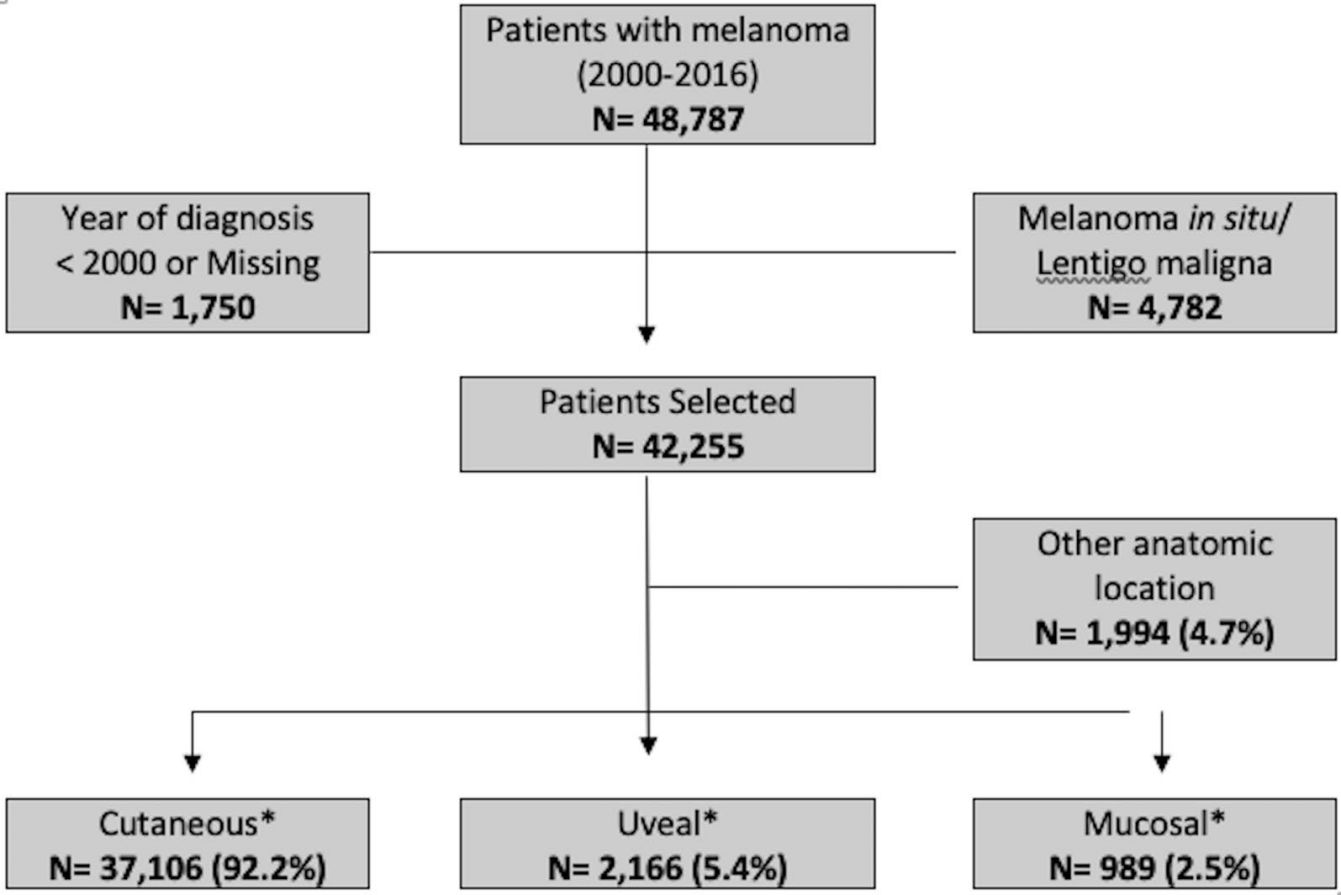
Flow chart of patient selection for the study

According to the International Classification of Diseases for Oncology (ICD-O), third edition, primary cutaneous melanomas (histologic codes, 8720–8723, 8730, 8740–8744, 8761, 8770–8774, 8745 and 8780) of head and neck (C44.0-C44.4), trunk (C44.5), upper limbs/shoulders (C44.6), lower limbs/hips (C44.7), or other, including overlapping areas of skin and not otherwise specified (NOS) (C44.8-C44.9); and primary sites for mucosal melanomas according to ICD10, nasal and paranasal (C30), vulva, vagina and uterine cervix (C51-C53), lips, oral cavity and pharynx (C00-C14), anal and rectal (C20-C21), other mucosae including digestive organs (C15-C26), respiratory tract and intrathoracic organs (C30-C39), penis and other male urinary tract non-specified (C60), genitourinary tract (C64.9-C68), peritoneum and retroperitoneum (C48) were selected. From all cases of ocular melanoma (ICD-O morphology codes 8720–8790 and ICD10 C69.0-C69.9) identified, uveal melanomas were included in the study (C69.2, C69.3-C69.4) [3]. Cases of conjunctival melanomas (C69.0), cornea NOS (C69.1), lacrimal gland (C69.5) and overlapping lesion of eye and adnexa (C69.6, C69.8-C69.9) were also excluded from the study (included in other anatomic location along with some other melanomas – Fig. 1). All included patients had clinical ophthalmic and/or pathological diagnosis of uveal melanoma.

### Statistical analysis

For the statistical analysis, Statistical Package for Social Sciences (SPSS) software, version 24, was used. Descriptive analysis using the frequency distribution of the variables was performed. The normality of the data was tested using the Kolmogorov–Smirnov test. Continuous variables were compared using Student t-test. Chi square Test was used for comparison of the distribution of the categorical variables and missing data was excluded from the analysis. The differences were considered of statistical significance when p-value was lower than 0.05.

### Ethical approval

This study was approved by the Ethics in Human Research Committee of the National Cancer Institute of Brazil (INCA), Rio de Janeiro, Brazil (reference number 128/11, CAAE – 0104.0.007.000-11) and is in accordance with the Good Clinical Practice Guidelines.

## Results

Considering the Brazilian hospital-based cancer registries between 2000 and 2016, a total of 48,787 cases of melanoma were identified and 42,255 patients were selected for the analysis. Cases diagnosed before 2000 or without the data of diagnosis were excluded (N = 1750) as well as cases of melanoma in situ/lentigo maligna (N = 4782). Cutaneous melanomas were identified in 37,106 (92.2%) patients and mucosal melanomas accounted for other 989 cases (2.5% of the total cases). There were 2423 cases of ocular melanomas (ICD10 C69.0–C69.9) identified and 257 cases of were also excluded: conjunctival (209 patients), corneal NOS (22 patients), lacrimal gland (3 patients) and overlapping lesion of the eye and adnexa (23 patients). Uveal melanomas were identified in 2166 cases patients, consisting of 5.4% of the total melanoma cases and 89.4% of all ocular melanomas (Fig. 1).

**Fig. 2:**
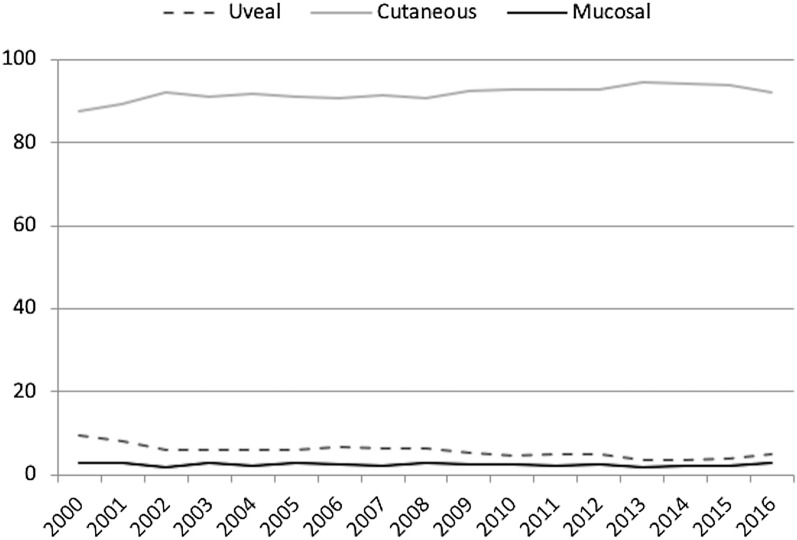
Proportional distribution of melanoma cases by year of diagnosis (N = 40,261)

Proportional distribution of uveal melanoma cases by year of diagnosis relating to cutaneous and mucosal melanomas may be seen in Fig. 2.

There was a slightly higher prevalence of uveal melanoma between women, 1139 cases (52.6%) in contrast with 1027 cases (47.4%) in men. The mean age was not statistically different (p = 0.408) between males (55.8 ± 14.8) and females (56.4 ± 15.2). Age distribution revealed 1411 cases (65.1%) in the group between 41 and 69 years old, 327 cases (15.1%) below 41 years old and 428 cases (19.8%) in patients with more than 70 years old of age.

Interestingly, there was an unequal regional distribution of uveal melanoma through the country, which did not respect the percentage of the total Brazilian population distribution. Regions Northeast, the second most populated area of the country accounting for 27.2% of the total Brazilian population, registered only 212 cases (9.8%); the North region, which accounts for 8.8% of the Brazilian population, registered 29 cases (1.3%) and the Center-West with 7.8% of the total Brazilian population, registered 21 cases (1.0%) [17].

Melanoma was localized in the choroid in 1440 cases (66.5%) and in the ciliary body and iris in 342 cases (15.8%). Other 384 cases (17.7%) were classified as uveal melanoma not otherwise specified (Table 1).

**Table 1:** Clinical stage at diagnosis prevalence according to patient characteristics of uveal melanoma in Brazil

Variables	Initial disease (stages I or II)	Metastases (regional or distant)	Missing/do not apply	Total	p value
	N^c^ (%^a^)	N^c^ (%^a^)	N^c^ (%^a^)	N^c^ (%^b^)	
Sex					0.607
Male	199 (19.4)	152 (14.8)	676 (65.8)	1027 (47.4)	
Female	230 (20.2)	182 (16.0)	727 (63.8)	1139 (52.6)	
Age (years)					0.214
≤ 40	66 (20.2)	52 (15.9)	209 (63.9)	327 (15.1)	
41–69	287 (20.3)	228 (16.2)	896 (63.5)	1411 (65.1)	
≥ 70	76 (17.8)	54 (12.6)	298 (69.6)	428 (19.8)	
Schooling (years)					*0.006*
< 8 years	179 (17.6)	174 (17.1)	662 (65.2)	1015 (46.9)	
≥ 8 years	113 (24.8)	73 (16.0)	270 (59.2)	456 (21.1)	
Missing	137	87	471	695 (32.1)	
Brazilian Region of the Hospital					* < 0.001*
North	2 (6.9)	3 (10.3)	24 (82.8)	29 (1.3)	
Northeast	21 (9.9)	27 (12.7)	164 (77.4)	212 (9.8)	
Center-West	1 (4.8)	1 (4.8)	19 (90.5)	21 (1.0)	
Southeast	393 (26.4)	272 (18.3)	821 (55.2)	1486 (68.6)	
South	12 (2.9)	31 (7.4)	375 (89.7)	418 (19.3)	
Topography					* < 0.001*
Choroid	370 (25.7)	273 (19.0)	797 (55.3)	1440 (66.5)	
Ciliary body and iris	52 (15.2)	42 (12.3)	248 (72.5)	342 (15.8)	
Uvea, NOS	7 (1.8)	19 (4.9)	358 (93.2)	384 (17.7)	
Total	429 (19.8)	334 (15.4)	1403 (64.8)	2166 (100.0)	

^a^Row percent

^b^Column percent

^c^Differences in total are due to missing data; percentages were calculated considering only patients with available data

Statistically significant associations are indicated in italics

A total of 429 (19.8%) patients were classified as initial disease and 334 (15.4%) as metastatic (regional or distant) based on TNM/AJCC Stage Groups. In 1403 (64.8%) patients the data of baseline staging was missing. Table 1 provides baseline characteristics and more detailed information about all uveal melanoma patients identified in the present study.

There were no differences in sex distribution and age at diagnosis considering initial (I and II) and advanced (regional or distant) stages. Diagnosis of initial disease was more frequent (113 cases–24.8%) in patients with more school years than in patients with less than elementary schooling only (179 cases–17.6%), p = 0.006. Distribution of initial disease and metastatic disease over the regions revealed that the southeast region registered 393 cases (26.4%) of initial disease and 272 cases (18.3%) of metastatic disease at presentation. The other regions demonstrated 77.4 to 90.5% of missing data regarding staging at site registration (Table 1).

The therapy offered for uveal melanoma treatment was also analyzed and differences in the total were due to missing data since they were calculated based on available data. Any treatment was provided to the patients in the vast majority of cases. In patients with initial disease, 402 (93.7%) received any treatment versus 27 (6.3%), which received none. On the other hand, in patients with metastases, 300 (89.8%) received any treatment versus 34 (10.2%), which received none. Overall, 1789 (83.1%) patients received treatment versus 365 (16.9%) that received none (p < 0.001). The main treatments were: surgery (47.3%) and radiotherapy (28.4%) (data not shown). Response to the first course of treatment was more frequent among initial disease patients (p < 0.001) (Table 2).

**Table 2:** Clinical stage at diagnosis prevalence according to diagnosis and treatment characteristics of uveal melanoma in Brazil

Variables	Initial disease (I or II)	Metastases (regional or distant)	Missing/do not apply	Total	p value
	N^c^ (%^a^)	N^c^ (%^a^)	N^c^ (%^a^)	N^c^ (%^b^)	
Year of diagnosis^d^					0.680
2000 a 2012	356 (20.1)	273 (15.4)	1138 (64.4)	1767 (81.6)	
2013 a 2016	73 (18.3)	61 (15.3)	265 (66.4)	399 (18.4)	
Time from diagnosis to treatment					*0.005*
≤ 60 days	236 (24.7)	183 (19.2)	535 (56.1)	954 (59.4)	
> 60 days	150 (23.0)	89 (13.7)	412 (63.3)	651 (40.6)	
No treatment	27	34	304	365	
Missing	16	28	152	196	
Response to the first course of treatment^e^					* < 0.001*
Response	43 (11.9)	28 (7.8)	290 (80.3)	361 (76.8)	
No response	4 (3.7)	29 (26.6)	76 (69.7)	109 (23.2)	
No treatment	27	34	304	365	
Missing	355	243	733	1331	
Total	429 (19.8)	334 (15.4)	1403 (64.8)	2166 (100.0)	

^a^Row percent

^b^Column percent

^c^Differences in total are due to missing data; percentages were calculated considering only patients with available data

^d^According to the federal law of 60 days that came into effect in 2013

^e^Response: partial remission, stable disease, and complete response; no response: progressive disease, relapsed disease or death

Statistically significant associations are indicated in italics

No change in staging at presentation after the Brazilian “60 days law” approval that came into effect in 2013 (a Federal law that regulates the maximum period that a patient with cancer has to wait, in order to initiate the treatment) was observed (p = 0.680) (Table 2).

## Discussion

To the best of our knowledge, this is the first study evaluating patients’ demographics regarding uveal melanoma in Brazil. Compared to cutaneous melanomas, uveal melanomas are much less prevalent, representing only 5.4% of melanomas in the current study. Also, the proportional distribution of cutaneous, uveal and mucosal melanoma cases per year had a small change yearly. It should be noticed that uvea is the far more common location of primary ocular melanoma. In this series 2423 cases of ocular melanomas were identified and 257 cases were classified as non-uveal ocular melanomas (10.6%); uveal melanomas represented 89.4% of the cases of ocular melanomas.

Similarly, Chang et al*.* described 82,943 cases of melanoma with a known primary site reported to the National Cancer Database (NCDB) in the United States between 1985 and 1994 and noticed that 5.5% (N = 4522) of melanomas were ocular, the second highest percentage. It was also noted that 85% of all ocular melanomas were classified as uveal melanomas (N = 3846), 4.5% of all melanoma cases.

Based in the same methodology of hospital-based epidemiology and searching the ICD for oncology coding, Singh et al*.* published data based on ﻿the Surveillance, Epidemiology and End Results (SEER) program of the National Cancer Institute for a 36-year period (from 1973 to 2008). They found 4999 cases of ocular melanoma and 4070 cases of uveal melanoma. An overall age-adjusted incidence of 5.1 cases per million population was also noted. Only 73% of those patients had histological confirmation of uveal melanoma [3]. Recently, Xu et al*.* published data based also on the SEER database and same methodology, although they have focused on incidence and survival outcomes. The authors reported an incidence of 4.64 per million population of uveal melanoma between 2010 and 2015 period and a significantly increasing annual percent changes of 4.22%, but did not mention any regional differences in prevalence and incidence data throughout the United States. They have also suggested that age at diagnosis, radiation therapy and American Joint Committee on Cancer (AJCC) stage may be potential predictors of prognosis [18].

Using data according to ICD9 topography codes 190.0 (iris and ciliary body), 190.5 (retina), and 190.6 (choroid) and ICD-O third edition for histologic codes 8720 to 8780 from the European Cancer Registry database (EUROCARE), from 67 cancer registries covering a combined population of 100 million people in 22 European countries, Virgili et al. identified 7051 ocular melanomas in the period of 1983 to 1994. Uveal melanomas accounted for 5566 cases (78.9%) [19].

Following an improvement of radiotherapy techniques worldwide, Mahendraraj et al*.* using SEER database between 1973 and 2012 revealed that more than two thirds of uveal melanoma patients are successfully treated with radiotherapy instead of surgery, resulting in an overall survival and cancer-specific survival improvement. Notwithstanding, the mean 5-year cancer-specific survival rate remained stable during the study period [20].

An ophthalmological center in Northern Brazil published the epidemiology of their first 3-year experience and from 71 cases of eye cancer, with 8 reported cases of uveal melanoma [21]. Another Brazilian study reported 269 orbital tumors with 6.7% of malignant melanomas of the orbital cavity, although their focus was on management of these patients and those could be not considered uveal melanomas [22].

Considering schooling, the difference in staging at the diagnosis being initial disease is more frequent in patients with more school years than in patients with less than elementary schooling only, and could reflect disparities in healthcare access between those two strata. A Polish study assessed the influence of one's education on the recognition of second primary cancers in uveal melanoma patients revealing that those with secondary and higher education had significantly more frequent diagnoses of second primary cancers than those who had primary and vocational education [23]. Meanwhile, Zinkhan et al*.* suggested that a higher socio-economic level is associated with a decreased risk for uveal melanoma [24]. Nevertheless, another German research group inferred that highest school degree or post-school professional degree and a two-dimensional social index show no association with uveal melanoma [25].

Uveal melanoma was mainly diagnosed in Southeast Brazil due to the highest concentration of citizens allied to the presence of the most important tertiary cancer centers with trained staff and equipment to provide diagnosis and management of the disease. In addition, Mierzwa-Dobranowska and Romanowska-Dixon have shown that residents of large cities usually present higher recognition of second primary cancers in uveal melanoma patients compared to their control group [26].

The Sistema Único de Saúde (SUS), the Brazilian Public Health System is the main health provider of a country with continental dimensions and more than 210 million inhabitants. Access to healthcare is extremely heterogenous in Brazil and can also explain the concentration of cases in a single geographic region. This also could explain why regions such as Northeast, the second most populated area of the country accounts for 27.2% of the total Brazilian population registered 212 cases (9.8%), North which accounts for 8.8% of the population registered 29 cases (1.3%) and Center-West with 7.78% of the population registered 21 cases (1.0%). It should be noticed that initial staging was missing from 55.2% to 90.5% of the cases [17].

The “Law of 60 days” was enforced in 2012 by the Brazilian Federal Government as a result of the long delays between the diagnosis and the initiation of cancer treatment nationwide. The Federal Law number 12.732/12 came into effect in 2013 in an attempt to accelerate the beginning of treatment after diagnosis and minimize the consequences of the huge delay between diagnosis and management of cancer. Unfortunately, after the implementation of the 60 days law, there was no difference in terms of diagnosis, staging and treatment, with the persistence of general difficulties in healthcare access such as long queues for patients who need assistance and delays in scheduling medical appointments [27].

Some limitations of the current study must be addressed. Missing data was relevant and may be explained by incorrect use of the database by the health professionals involved on data collection and handling, the complexity of the disease, and by the retrospective design using records collected over a long period of time. The cohort analyzed did not represent the entire Brazilian population; given this was a hospital-based registry study. Thus, such studies are more prone to selection bias than those based on population-based registries. Another limitation of this study was the lack of data available on secondary database to investigate the survival aspects of uveal melanoma in Brazil.

## Conclusion

Epidemiological data is critical for planning early treatment strategies and allocating medical resources in order to establish an efficient, democratic and homogeneous public healthcare. Because of the lack of epidemiological data, excessive missing information and possible under-notification, accurate measurement of incidence, prevalence and characteristics of uveal melanoma in the population is difficult, which may lead to hurdles in understanding the exact portrait of uveal melanoma in Brazil. This study intended to start closing this gap in ocular oncology. Nevertheless, further studies are required to clarify the uveal melanoma epidemiology not only in Brazil but worldwide. Advances in uveal melanoma research with regards to the molecular aspects of uveal melanoma are advancing faster than basic epidemiology with promising responses to immunotherapy and target therapy in metastatic disease.

## References

[1] McLaughlin CC, Wu XC, Jemal A, Martin HJ, Roche LM, Chen VW (2005). Incidence of noncutaneous melanomas in the U.S.. Cancer..

[2] Chattopadhyay C, Kim DW, Gombos DS, Oba J, Qin Y, Williams MD (2016). Uveal melanoma: From diagnosis to treatment and the science in between. Cancer.

[3] Singh AD, Turell ME, Topham AK. Uveal melanoma: Trends in incidence, treatment, and survival. Ophthalmology [Internet]. 2011;118(9):1881–5. Available from: http://dx.doi.org/10.1016/j.ophtha.2011.01.040 10.1016/j.ophtha.2011.01.040 21704381

[4] Singh AD, Bergman L, Seregard S (2005). Uveal melanoma: Epidemiologic aspects. Ophthalmol Clin North Am.

[5] Egan KM, Seddon JM, Glynn RJ, Gragoudas ES, Albert DM (1988). Epidemiologic aspects of uveal melanoma. Surv Ophthalmol..

[6] De Melo AC, Wainstein AJA, Buzaid AC, Thuler LCS (2018). Melanoma signature in Brazil: Epidemiology, incidence, mortality, and trend lessons from a continental mixed population country in the past 15 years. Melanoma Res.

[7] Cohen Goldemberg D, de Melo AC, de Melo Pino LC, Thuler LCS (2020). Epidemiological profile of mucosal melanoma in Brazil. Sci Rep.

[8] Instituto Nacional do Câncer. Estimate/2020 - Cancer incidence in Brazil. 2020. 300 p. https://www.inca.gov.br/sites/ufu.sti.inca.local/files//media/document//estimativa-2020-incidencia-de-cancer-no-brasil.pdf

[9] Ferrari NM, Muller H, Ribeiro M, Maia M, Sanches JA (2008). Cutaneous melanoma: Descriptive epidemiological study. Sao Paulo Med J.

[10] Schmerling RA, Loria D, Cinat G, Ramos WE, Cardona AF, Sánchez JL (2011). Cutaneous melanoma in Latin America: the need for more data. Rev Panam Salud Publica..

[11] Singh AD, Damato B, Howard P, Harbour JW (2005). Uveal melanoma: genetic aspects. Ophthalmol Clin North Am.

[12] Abdel-Rahman MH, Sample KM, Pilarski R, Walsh T, Grosel T, Kinnamon D (2020). Whole exome sequencing identifies candidate genes associated with hereditary predisposition to uveal melanoma. Ophthalmology..

[13] Zimmehman LE, McLean IW. Do Growth and Onset of Symptoms of Uveal Melanomas Indicate Subclinical Metastasis? Ophthalmology [Internet]. 1984;91(6):685–91. Available from: https://www.sciencedirect.com/science/article/pii/S0161642084342439 10.1016/s0161-6420(84)34243-9 6462627

[14] Damato B. Does ocular treatment of uveal melanoma influence survival? Br J Cancer [Internet]. 2010;103(3):285–90. Available from: http://dx.doi.org/10.1038/sj.bjc.6605765 10.1038/sj.bjc.6605765 PMC2920019 20661247

[15] Furdova A, Slezak P, Chorvath M, Waczulikova I, Sramka M, Kralik G (2010). No differences in outcome between radical surgical treatment (enucleation) and stereotactic radiosurgery in patients with posterior uveal melanoma. Neoplasma.

[16] Amin MB, Greene FL, Edge SB, Compton CC, Gershenwald JE, Brookland RK, et al. The Eighth Edition AJCC cancer staging manual: continuing to build a bridge from a population-based to a more “personalized” approach to cancer staging. CA Cancer J Clin. 2017;67(2):93–9. 10.3322/caac.21388 28094848

[17] IBGE. Censo Demográfico 2010, Características da População e dos Domicílios, Resultados do Universo. https://biblioteca.ibge.gov.br/visualizacao/periodicos/93/cd_2010_caracteristicas_populacao_domicilios.pdf. 2011.

[18] Xu Y, Lou L, Wang Y, Miao Q, Jin K, Chen M, et al. Epidemiological study of uveal melanoma from US surveillance, epidemiology, and end results program (2010–2015). J Ophthalmol. 2020;2020. 10.1155/2020/3614039 PMC7049826 32148939

[19] Virgili G, Gatta G, Ciccolallo L, Capocaccia R, Biggeri A, Crocetti E, Lutz JM, Paci E (2007). Incidence of Uveal Melanoma in Europe. Ophthalmology..

[20] Mahendraraj K, Lau CSM, Lee I, Chamberlain RS (2016). Trends in incidence, survival, and management of uveal melanoma: A population-based study of 7,516 patients from the surveillance, epidemiology, and end results database (1973–2012). Clin Ophthalmol.

[21] Cohen S, Pretyman CS, Sant’ana R, Singh N, Morales M, Belfort RN (2019). The Amazon Ocular Oncology Center: The first three years Centro de Oncologia Ocular do Amazonas: primeiros três anos. Arq Bras Oftalmol..

[22] Sirianni D, Leles CR, Mendonça EF (2013). A 12-year retrospective survey of management of patients with malignant neoplasms in the orbital cavity in a Brazilian Cancer Hospital. Open Dent J.

[23] Mierzwa-Dobranowska M, Romanowska-Dixon B (2012). Assessment of the influence of one’s education on early diagnosis of multiple primary cancer in patients with uveal melanoma. Klin Oczna.

[24] Zinkhan M, Stang A, Jöckel KH, Marr A, Bornfeld N, Schmidt-Pokrzywniak A (2013). Having children, social characteristics, smoking and the risk of uveal melanoma: A case-control study. Ophthalmic Epidemiol.

[25] Stang A, Ahrens W, Anastassiou G, Jöckel KH. Phenotypical characteristics, lifestyle, social class and uveal melanoma. 2003. 10.1076/opep.10.5.293.17319 14566630

[26] Mierzwa-Dobranowska M, Romanowska-Dixon B (2012). One’s location of residence as an important factor related to the occurrence of multiple primary cancer among patients with uveal melanoma. Klin Oczna.

[27] Paulino E, de Melo AC, Nogueira-Rodrigues A, Thuler LCS (2018). Gynecologic cancer in Brazil and the law of sixty days. J Gynecol Oncol.

